# Development of a seedling inoculation technique for rapid evaluation of soybean for resistance to *Phomopsis longicolla* under controlled conditions

**DOI:** 10.1186/s13007-018-0348-x

**Published:** 2018-09-11

**Authors:** Shuxian Li

**Affiliations:** Crop Genetics Research Unit, United States Department of Agriculture, Agricultural Research Service (USDA, ARS), P. O. Box 345, Stoneville, MS 38776 USA

**Keywords:** Soybean, *Phomopsis longicolla*, Phomopsis seed decay, Seedling inoculation, Resistance

## Abstract

**Background:**

Phomopsis seed decay (PSD) of soybean (*Glycine max* L. Merr.) is caused primarily by the seed-borne fungal pathogen *Phomopsis longicolla* T. W. Hobbs. The PSD disease reduces seed quality and yield worldwide. Development of effective techniques to evaluate soybean for resistance to PSD can facilitate identification of new sources of host resistance to manage the disease. This study was undertaken to develop and utilize a rapid cut-seedling inoculation technique to evaluate soybean genotypes for resistance to *P. longicolla* under controlled conditions.

**Results:**

There were no significant differences in stem lesion length determined as the area under disease progress curve at 24 °C and 30 °C. The 21 and 14-day-old seedlings were more susceptible than the older seedlings. Inoculation with 7 or 14-day-old pathogens caused higher values of AUDPC than older pathogen cultures. Isolates MS17-1 was the most aggressive isolate from the test of 25 isolates from seven states in the U.S. Eighteen previously reported field PSD-resistant accessions had significantly lower AUDPC than the susceptible checks and other entries (*P* ≤ 0.05).

**Conclusion:**

This study provided rapid evaluation of soybeans for reaction to *P. longicolla* and identification of PSD-resistant genotypes. Although PSD is a soybean seed disease, results from the cut-seedling inoculation assays without waiting a whole growing season were comparable to those obtained from field tests. Additionally, concerns about the environmental effects and uneven distribution of the pathogen in the field were ameliorated. The cut-seedling inoculation technique can also be used to speed up evaluation of PSD populations for the discovery of PSD-resistance gene(s), and high throughput phenotyping of seed diseases at seedling stage for genetics and genomic studies.

## Background

Soybean (*Glycine max* (L.) Merrill) is one of the most important economic crops in the world. With excellent sources of high protein and oil in the seeds, soybean is of global significance. At least 70 countries grew soybean in 2015 [1]. Global production of soybean was over 340 million metric tons in 2017 [2]. However, soybean production was suppressed by many diseases. Over 200 soybean pathogens have been identified, of which, approximately 35 are believed to be of economic importance [1].

Phomopsis seed decay (PSD) is one of the most economically important soybean diseases resulting in poor seed quality in most soybean-growing countries [3]. It is widespread and common in the mid-south region of the United States [4, 5]. PSD also severely reduces seed germination, seedling vigor, and stand establishment [5, 6]. This disease has caused significant economic losses in soybean [7]. Suppression of annual soybean yield caused by PSD in the United States has been as high as 0.38 million metric tons (MMT) with a total of 2.04 MMT loss from 1996 to 2007 [8]. Hot and humid environments favor PSD development and the growth of the causal pathogen *Phomopsis longicolla* T.W. Hobbs during seed fill and maturity in the southern United States consequently, in 2009. Soybean losses due to PSD in 16 southern states of United States were over 0.33 MMT [9]. Nationally, PSD resulted in yield loss with average of about 0.2 MMT from 1996 to 2014 but in 2009, losses were estimated over 0.6 MMT (http://extension.cropsci.illinois.edu/fieldcrops/diseases/yield_reductions.php). Although PSD occurs throughout the soybean production area, its incidence and severity varies year by year, particularly in relation to the weather during late soybean growth season [10, 11]. Delayed harvest can result in high incidences of PSD in wet and warm environments [12, 13].

Although *P. longicolla* is a seed-borne pathogen, it can infect any part of soybean at any growth stage [4, 14]. However, seeds are most susceptible to PSD, especially when plants reach the R7 growth stage (beginning maturity, one normal pod on the main stem has reached its mature pod color) or physiological maturity [5, 15]. Soybean seed infected by *P. longicolla* can either have symptoms with cracked seed coats or discoloration, or no visible symptoms [16, 17].

PSD has been reported to be managed by several methods, such as crop rotation to non-host crops, conventional tillage to reduce inoculum and spore dissemination by *P. longicolla*, and foliar fungicide treatments at soybean reproduction growth stages [10, 18–21]. However, these practices do not consistently and effectively reduce PSD. Harvesting mature seeds on time could reduce PSD, but rainfall often delays harvests. Use of host genetic resistance and planting PSD-resistant soybean remains the most economically and environmentally beneficial way of reducing losses to PSD in soybean [22–27]. Moreover, resistant cultivars can provide disease protection at no additional cost to the grower above the price of the planting seed. However, very few cultivars have been found to be completely resistant to PSD.

Field screening of soybean accessions from the USDA Germplasm Collection (http://www.ars-grin.gov/npgs/) and commercial soybean cultivars for resistance to PSD have been reported in the past decades. Experiments were conducted to screen soybeans in Missouri and Puerto Rico from 1983 through 1985. ‘PI 417479’ was identified as highly resistant to PSD [28]. In a 4-year project from 2007 to 2010, seeds of 208 representative maturity group (MG) V soybean plant introductions (PIs) were field-tested in Stoneville, Mississippi and eight new sources of resistance to PSD disease were discovered [29]. Over a 5-year period, 135 soybean germplasm entries representing 28 geographic origins with MG III to V were screened for resistance to PSD. Field tests were conducted in Arkansas, Mississippi, and Missouri of the U.S., and 23 new sources of PSD-resistance were identified [12]. In addition, reactions of soybean commercial cultivars and breeding lines were also evaluated [13, 30]. In those field experiments, the traditional seed plating assay could not be performed until seeds were harvested from the field. It is a long and time-consuming process. Hence, development of a rapid disease evaluation technique that can be used earlier in plant development than the traditional seed plating assay is needed to facilitate identification of new sources of host resistance for managing PSD. Since *P. longicolla* can infect all parts of soybean tissues at any growth stages [17], it was hypothesized that results from inoculation of soybean seedlings under controlled conditions and the measurement of stem lesions after inoculation would be comparable to the seed assay from the whole season derived field tests without concern of environmental effects.

Hence, the objectives of this research were to: (1) develop a cut-seedling inoculation technique under controlled conditions to evaluate soybean for resistance to *Phomopsis longicolla*; (2) apply the cut-seedling inoculation technique to test selected soybean entries from our previous field trials at Stoneville, Mississippi; and (3) analyze the correlation between the values of area under disease progress curves (AUDPCs) of stem lesion length from cut-seedling inoculation experiments and the percentage of *P. longicolla* seed infection from previous field trials. Outcomes of this research will facilitate identification of genotypes with resistance to PSD and aid in development of PSD-resistant soybean lines or cultivars for the breeding program.

## Methods

### Description of plant materials and planting

A PSD-susceptible soybean cultivar, ‘Williams 82’, was used in the experiments to test effects of plant age, pathogen culture age, different isolates, and temperature on the stem lesion length caused by *P. longicolla.* In addition, a total of 42 soybean entries (14 of each MG III, IV, and V) including 36 plant introductions (PI) and six cultivars (AP 350, IA3001, SUWEON97, TARA, Williams 82, and 5002T) were used in this study. Those PIs originated from 16 countries representing diverse set of the origins and/or commercial production areas in the USDA Soybean Germplasm Collection [12]. Soybean cultivars tested were originally from the USA, except cultivar SUWEON97 which is originally from South Korea. Cultivar 5002T is a conventional cultivar for the southern USA and a yield check for the USDA Uniform Soybean Test (http://www.ars.usda.gov/SP2UserFiles/Place/60661000/UniformSoybeanTests/2013SoyBook.pdf). All soybean seed were obtained from the USDA Soybean Germplasm Collection in Urbana, IL. The host reaction of IA3001, SUWEON97, TARA, and 5002T to *P. longicolla* were unknown and AP 350 and Williams 82 were susceptible [31, 32].

Experiments were conducted at the USDA, ARS, Jamie Whitten Delta States Research Center in Stoneville, Mississippi. Five to seven seeds of each soybean entry were over sown in 10-cm-diameter pots containing Sun Grow Metro Mix 360 soil (Sun Grow Horticulture Products, Belleview, WA). Plants in each pot were thinned to three plants before inoculation. Two to three extra pots of each soybean entry also were planted to allow adequate plants for the test. Before inoculation, pots were re-arranged in plastic trays with size of 42.4 × 27.4 cm (Rubbermaid, Huntersville, NC) based on the experimental design. Plants were placed in the Conviron PGR 15 growth chamber (Conviron Inc. Winnipeg, Canada) under a 16-h photoperiod with a light intensity of 433 μE m^−2^ s^−1^ at 24 °C. The humidity set point was 90%.

### Pathogen isolates of *Phomopsis longicolla*

A total of 25 fungal isolates (24 *P. longicolla* and one *Diaporthe aspalathi*) were tested in this study. They were isolated from soybean seeds harvested from fields in seven states including Arkansas, Illinois, Maryland, Missouri, Mississippi, Ohio, and Virginia. Isolate OH 83-T is the type strain TWH P74 (ATCC 60325) obtained from the American Type Culture Collection(ATCC), which was originally isolated by Hobbs et al. [14] in Ohio and maintained in USDA, ARS research facility at Stoneville, Mississippi [33]. Isolate MSPL 10-6 of *P. longicoll*a was originally from field-grown soybean plants in Stoneville, Mississippi, and has also been used in previous studies to identify new sources of resistance to PSD [12, 13, 29] and genomic study [34]. The *D. aspalathi* isolate MS14-1 (MS-SSC91) has been used to evaluate soybean for resistance to stem canker as part of the USDA Uniform Soybean Tests (http://www.ars.usda.gov/SP2UserFiles/Place/60661000/UniformSoybeanTests/2013SoyBook.pdf) [35]. Because isolate MSPL 10-6 was one of the most aggressive isolates in early preliminary greenhouse tests (data not shown), it was selected to use for the cut-stem inoculation to test effects of plant age, pathogen culture age, and temperature on the stem lesion length caused by *P. longicolla*. All isolates were grown at 24 °C on triplicated Petri dishes (100 mm in diameter) of acidified potato dextrose agar (Difco Laboratories, Detroit, MI) adjusted to pH 4.8 with 25% (w/v) lactic acid (APDA) after autoclaving, transferred periodically as needed and maintained at 4 °C for further use or stored in 30% (v/v) glycerol in a − 80 °C freezer. Inoculation with fungal-free ADPA plug was the negative control for experiments.

### Inoculum preparation and the cut-seedling inoculation technique

**Fig. 1:**
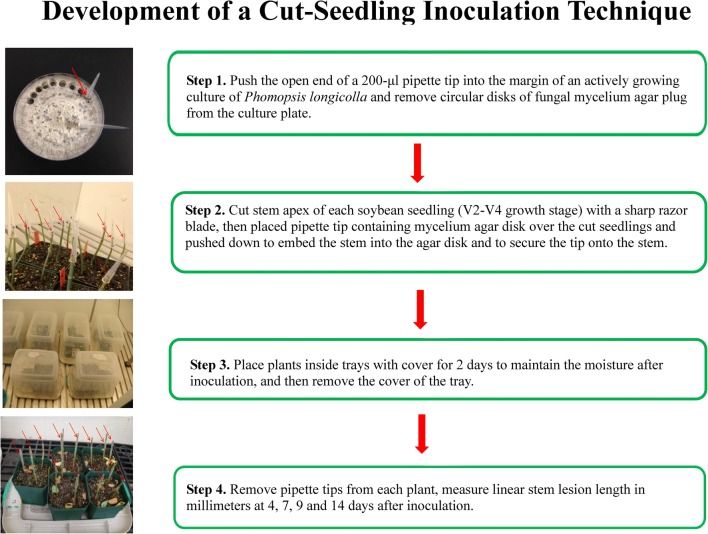
The cut-seedling inoculation techniques for evaluating soybean for resistance to *Phomopsis longicolla*

To prepare the inoculum, isolates of *P. longicolla* were grown at 24 °C on APDA. The culture ages were from 1 to 6 weeks old depending on the experiments. For inoculation, the stem apex of each soybean seedling (V2-R2 growth stage) was cut 25 mm above the unifoliolate node with a sharp razor blade. The open end of a 200-μl clear pipette tip (Daigger, Vernon Hill, IL) was pushed into the margin of an actively growing *P. longicolla* culture. Circular disks of fungal mycelium agar plug (0.5 mm in diameter) was cut and removed from the culture plate. The pipette tip containing the agar disk with *P. longicolla* mycelium was immediately placed over the cut part of the seedling and pushed down to embed the stem into the agar disk and to secure the tip onto the stem. Plants were placed inside plastic trays with covers for 2 days to maintain the moisture after inoculation. Thereafter, the pipette tips were removed from each plant. Linear stem lesion length in millimeters was measured at 4, 7, 11, and 14 days after inoculation. The general procedure for the cut-seedling inoculation technique is illustrated in Fig. 1.

### Experiment to test effect of plant age on stem and stem lesion lengths

Seedlings of Williams 82 at 14, 21, 28, and 35 days old were arranged in a randomized complete block design with 4 replications. Within each block, there were two levels of subsamples: 3 pots per each treatment (plant age) and 3 plants per pot. The experimental unit was a set of 3 pots. There were 9 plants for each plant age tested in each trial. Inoculation was performed with a 14-day-old culture of MSPL 10-6 agar plug as inoculum on the same day when four different age plants (14, 21, 28, and 35 days old) were available. Treatment with APDA agar plugs without fungus was the negative control. Stem length and stem lesion length were measured at 4, 7, 11, and 14 days after inoculation. Plant growth condition and the cut-seedling inoculation method have been described above. The experiment was repeated twice.

### Experiment to test effect of pathogen age on stem and stem lesion lengths

Local *P. longicolla* isolate MSPL 10-6 with six culture ages of 7, 14, 21, 28, 35, and 42 days old (1–6 weeks) was used to inoculate 21-day-old seedlings of Williams 82. Treatment with APDA agar plug without fungus was the negative control. Plants were arranged in a randomized complete block design with three replications. Within each block, there were 2 levels of subsamples: 3 pots per each treatment (6 different ages of pathogen) and 3 plants per pot. The experimental unit was a set of 3 pots. There were 9 plants of each isolate age tested in each trial. Plants were grown in the Conviron PRG15 growth chamber at 24 °C. The cut-seedling inoculation was performed using pipette tip containing the agar disk with *P. longicolla* mycelium as described in the section of “inoculum preparation and the cut-seedling inoculation technique” above. Stem length and stem lesion length were measured at 4, 7, 11, and 14 days after inoculation. The experiment was repeated twice.

### Experiment to test effect of post-inoculation temperature on stem and stem lesion lengths

Soybean Williams 82 were planted and grown at 24 °C for 3 weeks. Plants were then placed into two Conviron PRG15 growth chambers; one chamber was set to 24 °C and another at 30 °C. All 3-week-old seedlings of Williams 82 were inoculated with a 14-day-old cultural agar plug of a *P. longicolla* isolate MSPL 10-6. Treatment with APDA agar plug without fungus was the negative control. Plants were arranged in a randomized complete block design with five replications. Within each block, there were 2 levels of subsamples: 5 pots per each treatment (24 and 30 °C) and 3 plants per pot. The experimental unit was a set of 5 pots. There were 15 plants of each post-inoculation temperature tested in each trial. Stem length and stem lesion length were measured at 4, 7, 11, and 14 days after inoculation. The experiment was repeated three times.

### Experiment to test effects of different pathogen isolates on stem lesion lengths

Twenty-five fungal isolates including 24 *P. longicolla* and one *D. aspalathi* from seven states were used to inoculate 21-day-old seedlings of Williams 82. Treatment with APDA agar plug without fungus was the negative control. Plants were arranged in a randomized complete block design with three replications. Within each block, there were 2 subsamples: 3 pots per each isolate treatment (25 different isolates); and 3 plants per pot. The experimental unit was a set of 3 pots. There were 9 plants of each isolate tested in each trial. Stem length and stem lesion length were measured at 4, 7, 11, and 14 days after inoculation. Plant growth condition and the cut-seedling inoculation method were described previously. The experiment was repeated once.

### Experiments to evaluate soybean for resistance to *Phomopsis longicolla*

Three experiments for each of three maturity groups (MG III, IV, and V) of soybean were conducted with 14 entries in each maturity group. All 21-day-old seedlings of each entry were cut and inoculated with a 2-week-old cultural agar plug of a *P. longicolla* isolate MSPL 10-6. Soybean entries were arranged in a randomized complete block design with three replications. Within each block, there were 2 levels of subsamples: 3 pots per each entry and 3 plants per pot. The experimental unit was a set of 3 pots. There were 9 plants of each entry tested in each trial for each experiment. Stem lesion length were measured at 2, 5, and 7 days after inoculation. Inoculated seedlings were cut at 7 days after inoculation and dried at 55 °C in an oven (Model 6905, Fisher Scientific, Memphis, Tennessee) for two days and then weighed. The experiment was repeated once.

### Data analysis

Data for the negative control, in which plants were treated with APDA plug without the fungal pathogen and did not show stem lesion, were removed prior to data analysis to avoid violating homogeneous variance assumptions. Area under growth progress curve (AUGPC) and area under disease progress curve (AUDPC) were based on visual measurement of stem length and stem lesion length from 4 to 14 days after inoculation of soybean and was calculated by trapezoidal integration [36].

Data were averaged on subsamples within each block and combined over trials for each experiment. Analyses of variance was performed using the generalized linear mixed model (PROC GLMMIX) of SAS (Statistical Analysis System, version 9.4, SAS Institute, Cary, NC) with random effects for Trial, Block within Trial, and Treatment × Trial.

For the experiments evaluating three maturity groups of soybean, entries were compared with least significant difference (LSD) at *P* ≤ 0.05. The PROC CORR procedure of SAS was used to compute Pearson’s correlation coefficients between AUDPC values of stem lesion length and dry weight from cut-seedling inoculation experiments and the percentage of *P. longicola* seed infection from previous field trials.

## Results

### Effects of plant age on stem and stem lesion lengths

Results of ANOVA analysis show that there were significant (*P* = 0.0296) differences among the plant ages at inoculation with different growth stages for AUDPC values (Table 1). The 21 and 14-day-old seedlings had greater AUDPC values than the older seedlings at 28 and 35 days old (Table 2). However, there were no significant (*P* > 0.05) differences in AUGPC values for the stem length of plants at 21, 28, and 35 days old except that 14-day-old plants that had lower AUGPC values.

**Table 1:** Analysis of variance of experiments for evaluating seedling cut-stem inoculation method and soybean genotypes for resistance to Phomopsis seed decay based on the area under disease progress curve (AUDPC) values for stem lesion length

Experiment	Treatment effects	Num DF^a^	Den DF^b^	*F* value	*P* ≥ F
1	Plant age at inoculation	3	41	3.30	0.0296
2	Fungal age at inoculation	5	6	3.19	0.0969
3	Temperature	1	3	0.94	0.4039
4	Isolate	25	16	12.80	< 0.0001
5	Soybean genotype MG III	13	13	4.69	0.0045
6	Soybean genotype MG IV	13	13	5.78	0.0017
7	Soybean genotype MG V	13	14	4.62	0.0038

Analysis of variance was performed using the generalized linear mixed model (PROC GLMMIX) of SAS (version 9.4, SAS Institute, Cary, NC) with random effects for Trial, Block within Trial, and Treatment × Trial

^a^The numerator degrees of freedom

^b^The denominator degrees of freedom calculated based on Kendward and Rogers approximation method [37]

**Table 2:** Effect of plant age at inoculation with *Phomopsis longicolla* on stem length and stem lesion length in the seedling cut-stem inoculation experiments

Plant age at inoculation (days)	Growth stage*	Stem length (AUGPC)**	Lesion length (AUDPC)**
14	V2	828.9 b	257.5 a
21	V3	1394.3 a	266.9 a
28	V4	1240.3 a	198.6 b
35	R1	1300.1 a	119.5 b
Mean		1190.9	230.6

*Growth stage was determined as described by Fehr and Caviness [38]. V = vegetative growth stage and R = reproductive growth stage

**Means followed by the same letter within a column are not significantly different by the least significant difference test at *P* ≤ 0.05 as determined by the SAS GLMMIX procedure

### Effect of pathogen age on stem and stem lesion lengths

Although overall the AUDPC values of the pathogen ages tested were not significantly (*P* = 0.096) different (Table 1), the youngest culture of *P. longicolla* at 7-day-old (1-week old) caused the greatest AUDPC value, while pathogen cultures of 35 days and 42 days old had the lowest AUDPC values in the tests (Table 3). The pathogen culture ages did not affect the stem length in the cut-seedling inoculation tests. Plants inoculated with *P. longicolla* that were 7–42 days old (1–6 weeks) had the similar AUGPC values (Table 3).

**Table 3:** Effect of culture age of *Phomopsis longicolla* on stem length and stem lesion length of soybean cultivar Williams 82 in seedling cut-stem inoculation experiments

Culture age (week)	Stem length (AUGPC)*	Lesion length (AUDPC)*
1	695.8 a	194.3 a
2	673.9 a	159.9 ab
3	681.9 a	127.6 ab
4	704.2 a	130.0 ab
5	659.4 a	107.7 b
6	669.5 a	72.1 b
Mean	680.8	131.9

*Means followed by the same letter within a column are not significantly different by the least significant difference test at *P* ≤ 0.05 as determined by the SAS GLMMIX procedure

### Effect of temperature on stem and stem lesion lengths

There was no significant (*P* = 0.4039) differences of stem lesion length caused by *P. longicolla* at 24 °C and 30 °C (Table 1) although the mean values of AUDPC (391.5) at 30 °C seemed a little greater than that (329.0) at 24 °C (Table 4). Those two temperatures did not affect the stem length in the cut-seedling inoculation tests. Plants inoculated with *P. longicolla* in the growth chambers at either 24 °C or 30 °C had similar AUGPC values (Table 4).

**Table 4:** Effect of post-inoculation temperatures on stem length and stem lesion length of soybean cultivar Williams 82 inoculated with *Phomopsis longicolla* in the seedling cut-stem inoculation experiments

Temperature (°C)	Stem length (AUGPC)*	Lesion length (AUDPC)*
24	814.50 a	328.97 a
30	791.03 a	391.50 a

*Means followed by the same letter within a column are not significantly different by the least significant difference test at *P* ≤ 0.05 as determined by the SAS GLMMIX procedure

### Effect of different pathogen isolates on stem lesion lengths

Results of ANOVA analysis show that there were significant (*P* < 0.0001) differences among the isolates for the AUDPC values (Table 1). The range of the AUDPC values was from 10.00 to 372.50. Isolate of *P. longicolla* MS17-1 was the most aggressive isolate causing the highest values of AUDPC, while the *D. aspalathi* isolate (MS 14-1) had the lowest AUDPC value on Williams 82 seedlings in this study (Table 5). Plants treated with PDA without fungus did not exhibit stem lesions. They were excluded from the data analysis.

**Table 5:** Effect of *Phomopsis longicolla* isolates on stem lesion length of soybean cultivar Williams 82 in the cut-stem inoculation experiments

Isolate	Geographic origin	Year isolated	Lesion length (AUDPC)*
AR 09-1	Arkansas	2009	181.33 abc
IL 16-1	Illinois	2016	158.17 abc
IL 16-3	Illinois	2016	147.17 bc
IL 16-2	Illinois	2016	79.25 bc
MD 16-1	Maryland	2016	263.50 abc
MO 09-2	Missouri	2009	240.00 abc
MO 16-2	Missouri	2016	138.00 bc
MO 16-3	Missouri	2016	126.00 bc
MS 06-2	Mississippi	2016	280.00 ab
MS 08-1	Mississippi	2008	210.83 abc
MS 10-6SS	Mississippi	2010	208.75 abc
MS 14-1	Mississippi	2014	10.00 d
MS 14-15	Mississippi	2014	227.00 abc
MS 15-185	Mississippi	2015	188.44 abc
MS 15-80	Mississippi	2015	103.39 bc
MS 16-1	Mississippi	2016	234.06 abc
MS 16-2	Mississippi	2016	364.67 ab
MS 16-24B	Mississippi	2016	246.67 abc
MS 16-3	Mississippi	2016	265.50 ab
MS 17-1	Mississippi	2017	372.50 a
MS 17-36	Mississippi	2017	228.00 abc
OH 83-T**	Ohio	1983	97.67 bc
VA 16-1	Virginia	2016	182.67 abc
VA 16-31	Virginia	2016	287.83 ab
VA 16-40	Virginia	2016	267.50 ab
Mean			204.36

*Means followed by the same letter within a column are not significantly different by the least significant difference test at *P* ≤ 0.05 as determined by the SAS GLMMIX procedure

**Type strain TWH P74 (ATCC 60325) was obtained from the American Type Culture Collection (ATCC)

### Reaction of soybean genotypes to *Phomopsis longicolla*

Differences among genotype reactions to *P. longicolla* were found in all three experiments including MG III (*P* = 0.0045), MG VI (*P* = 0.0017) and MG V (*P* = 0.0038) with the cut-seedling inoculation technique (Table 1). The AUDPC values in the tests of MG III ranged from 9.77 to 49.57. The dry weights of three seedlings ranged from 0.95 to 3.29 grams (g). In the MG VI tests, the AUDPC values were from 13.00 to 44.54, the dry weights were 1.71 g to 3.68 g. The range of the AUDPC values in MG V was from 13.02 to 30.86, while the dry weight values were from 1.68 to 4.40 g (Table 6).

**Table 6:** Effect of soybean genotypes on stem lesion length after inoculation of soybean with mycelial plug of *Phomopsis longicolla* isolate of MS10-6 in the seedling cut-stem inoculation experiments

Entry	Origin	Maturity group	Lesion length (AUDPC)*	Dry weight (g)*
PI 504488**	Taiwan	III	9.77 d	3.29 a
PI 417361**	Japan	III	10.86 d	2.87 a
PI 88490**	China	III	13.51 cd	2.81 a
PI 189891**	France	III	14.25 cd	2.49 ab
PI 398697**	S. Korea	III	14.41 cd	2.53 ab
PI 504481**	Taiwan	III	14.58 cd	2.85 a
PI 437482	Russia	III	16.52 cd	2.22 abc
PI 398752	S. Korea	III	20.24 bcd	2.65 ab
PI 548298	Canada	III	22.87 bcd	2.39 ab
PI 547827	USA	III	23.32 bcd	2.49 ab
PI 578486	USA	III	28.84 bc	1.60 bcd
IA3001****	India	III	32.53 b	1.30 cd
PI416988	USA	III	35.60 ab	1.72 bcd
Williams 82***,****	Japan	III	49.57 a	0.95 d
Mean			21.92	2.3
PI 416779**	Japan	IV	13.00 f	3.68 ab
PI 235335**	Uruguay	IV	17.43 ef	3.91 a
PI 346308**	India	IV	17.59 def	3.01 bc
PI 158765**	China	IV	18.37 cdef	3.08 bc
PI 87074	S. Korea	IV	18.59 cdef	2.97 bcd
SUWEON97****	USA	IV	22.86 bcdef	2.20 bcd
PI 346307	India	IV	24.16 bcde	2.83 cdef
PI 404173	China	IV	24.78 bcde	2.97 bcd
PI 235346	Uruguay	IV	27.63 bcd	2.61 cdef
PI 355070	USA	IV	27.98 bc	2.60 cdef
PI 80479	Japan	IV	29.84 b	1.81 g
PI 264555	Argentina	IV	30.72 b	2.18 efg
PI 371611	Pakistan	IV	30.82 b	1.85 fg
AP 350***,****	USA	IV	44.54 a	1.71 g
Mean			24.88	2.67
PI 417567**	Taiwan	V	13.02 d	4.40 a
PI 381659**	Uganda	V	14.42 d	3.13 bcd
TARA****	USA	V	15.74 cd	3.28 bc
PI 407749**	China	V	15.94 cd	2.77 cde
PI 381668**	Uganda	V	16.19 cd	2.79 cde
PI 471938	Nepal	V	16.40 cd	3.20 bc
PI 476920**	Vietnam	V	16.72 cd	2.85 bcde
5002T****	USA	V	16.88 cd	3.54 b
PI 407752	China	V	18.61 cd	2.42 def
PI 506844	Japan	V	19.92 cd	2.65 cde
PI 417098	Japan	V	22.12 bc	2.35 efg
PI 417420	Japan	V	22.21 bc	1.77 fg
PI 172902	Turkey	V	27.15 ab	1.74 fg
PI 507690***	Russia	V	30.86 a	1.68 g
Mean			19.01	2.75

*Means followed by the same letter within a column and maturity group are not significantly different by the least significant difference test at *P* ≤ 0.05 as determined by the SAS GLMMIX procedure

**Reported resistant line/check

***Susceptible check

****Cultivar check

In the test of MG III genotypes, all six previously reported field PSD-resistant genotypes PI 189891, PI 398697, PI 417361, PI 504481, PI 504488, and PI 88490 had significantly (*P* ≤ 0.05) lower stem lesion length than the susceptible check Williams 82, as well as IA 3001 and PI 416988. PI 504488 had the lowest AUDPC value of 9.77, while Williams had the AUDPC value of 49.57 (Table 6).

In the MG IV tests, a previously reported field PSD-resistant genotype PI 416779 had the lowest stem lesion length among the genotypes tested. The AUDPC value for PI 416779 was 13.00 while the susceptible check AP 350 had the AUDPC value of 44.54. Other three resistant lines, PI 158765, PI 235335, and PI 346308, had significantly (*P* ≤ 0.05) lower stem lesion length than the susceptible check APS 350. In addition, PI 80479, PI 264555 and PI 371611 had susceptible reactions to *P. longicolla* (Table 6).

In the MG V tests, PI 507690 was the susceptible check, which had a significantly higher AUDPC value than all other 13 entries. Five resistant entries (PI 381659, PI 381668, PI 407749, PI 417567, and PI 476920) entries had significantly (*P* ≤ 0.05) lower stem lesion length than PI 507690 in the same test. Two cultivar checks, TARA and 50021T appeared to have moderately resistant reactions to *P. longicolla.*

The AUDPC values were significantly (*P* ≤ 0.001) negatively correlated with dry weight in all three maturity group genotype experiments with the cut-seedling inoculation technique (Table 7). Moreover, using the field data from our previous field experiments on those soybean entries [12], the AUDPC values of MG III genotypes were positively correlated with the percentage of seed infection with *P. longicolla* in the trial in 2009 with natural infection, as well as the 2012 field inoculated experiments. The AUDPC values of MG IV genotypes were significantly correlated with the field non-inoculated experiments in 2009, but not the field experiments in 2012. For the MG V genotypes, the AUDPC values were positively correlated with the percentage of *P. longicolla* seed infection in the 2012 field inoculated experiments. None of the AUDPC values of all three maturity group genotypes were significantly correlated with the non-inoculated trials in 2012 (Table 7).

**Table 7:** Pearson correlation coefficients between the area under disease progress curve (AUDPC) values of stem lesion length and dry weight from the cut-seedling inoculation experiments and the percentage of seed infection with *Phomopsis longicolla* in the field experiments

Maturity group	Dry weight		2009 field (Non)*		2012 field (Non)*		2012 field (Inoc)*	
	r**	P***	r**	P***	r**	P***	r**	P***
III	− 0.9242	< 0.0001	0.6231	0.0173	0.3481	0.2225	0.6598	0.0102
IV	− 0.8371	0.0002	0.5460	0.0434	0.0093	0.9750	0.4050	0.1508
V	− 0.8493	0.0001	0.2801	0.3321	0.0055	0.9851	0.7099	0.0045

Field data were from our previous field experiments [12]

*Field inoculation experiments. Non: Noninoculated trials, plants were sprayed with distilled water; Inoc: Inoculated with spore suspension of *P. longicolla* (2 × 10^5^) at the R5 stage

**Pearson correlation coefficients

***Probability

## Discussion

If adopted there are several clear advantages to the use of the cut-seedling inoculation technique. First, traditional approaches to evaluating a cultivar’s response to PSD involve seed assessments conducted at the end of the growing season. For soybean, this means waiting approximately 102–164 days [39] before a cultivar’s response to PSD is known. In this respect, the advantage of using the cut-seedling inoculation assay is that it can be conducted within 2–3 weeks of seedling establishment with the measurement of stem lesion length, which significantly shortens the time required to accurately screen for resistance to PSD.

A second advantage of the cut-seedling inoculation technique is that by conducting these assays under controlled conditions in a growth chamber, the results are not influenced by environmental factors. This is in contrast to field based assays that can be strongly influenced by fluctuating weather conditions such as humidity and temperature [16, 40–43]. For instance in 2009, environmental conditions were conducive to PSD, the natural *P. longicolla* seed infection (without inoculation treatment) was as high as 80% in field trials conducted in three southern states (AR, MO, and MS). However, in 2011, hot and dry weather, especially during the period from the pod fill through harvest stages, led to almost no seed infection [12]. The influence of varying environmental conditions in the field makes it difficult to screen soybean entries and identify durable PSD-resistant genotypes. With the cut-seedling inoculation techniques, all tests were conducted in environmentally controlled growth chambers thus ensuring that fluctuating environmental conditions do not influence the outcome of PSD. As a result, these controlled assays enable more accurate scoring of soybean cultivar sensitivity to PSD.

The third advantage of the cut-seedling inoculation technique is greater consistency with the starting inoculum, ensuring that every single seedling receives the same dose of *P. longicolla*. In contrast, assays conducted in the field suffer from uneven inoculation, with the potential for some soybean entries to survive *P. longicolla* infection if the inoculum was sufficiently low. Uneven inoculation therefore affects the accuracy of scoring for resistance to PSD in the field.

Many scientists have used inoculation treatments to increase disease pressure in field and greenhouse experiments for identification and confirmation of disease resistance in soybean [29, 44–46]. This approach could provide a more uniform distribution of the pathogen on soybean tested and reduce the chance of escapes, especially when the environmental conditions are not conducive for the disease development.

As pointed out by Li et al. [32], pathogen diversity could be another factor contributing to the differences of soybean entries in responding to the attack by *P. longicolla*. Different isolates of *P. longicolla* could cause different levels of infection on soybean. However, comparing isolates for aggressiveness based on seed infecting characteristics may not be practically possible in the field because there may be different isolates existing in the same field at the same time. It was reported that a set of selected 48 *P. longicolla* and *Phomopsis* spp. isolates that were collected both from soybean host or non-legume host, and from the USA, Canada and Costa Rica were evaluated in greenhouse using the cut-stem inoculation method. Aggressive isolates were identified [32]. Here, a different set of isolates were tested in growth chambers. Results clearly demonstrated the difference of the various isolates in their ability to infect soybean. Isolate MS17-1 was the most aggressive isolate from the test of 25 isolates. For breeding for resistance to PSD, it is critical to choose specific isolates that are highly pathogenic. With the cut-seedling inoculation in growth chamber, it is easy to measure stem length and stem lesion length under controlled conditions, and to provide quantitative measurements of the infection by isolates on soybean. In addition, results showed that Williams 82 was not susceptible to *D. aspalathi*. Both *P. longicolla* and *D. aspalathi* are the member of *Diaporthe*–*Phomopsis* complex. These two fungal species are taxonomically closed related with similar culture morphology [47]. However, *P. longicolla* is the primary agent of PSD while *D. aspalathi* causes stem cankers in soybean. Selection of the right host plant is therefore an important consideration when using the cut-seedling inoculation technique. Additionally, cultivar Williams 82 could be used to rapidly distinguish between these two fungal species rather than relying on the more laborious morphological and molecular approaches.

In this study, none of the AUDPC values of all maturity group entries were significantly correlated with the percentage of seed infection with *P. longicolla* in 2012 field non-inoculated experiments, but the AUDPC of MG III and MG V entries were significantly correlated with the percentage of *P. longicolla* seed infection in 2012 field inoculated experiments. It appears that inoculation treatment in the field could reduce the number of escapes due to the possible uneven distribution of the pathogen in the field. The AUDPC values of MG IV were significantly correlated with the field non-inoculation experiments in 2009, but not the experiments in 2012. In view of the field data for MG IV soybean trial, all four reported soybean entries had resistant reaction to PSD in both 2009 and 2012. However, some entries had completely different reaction to PSD [12]. For example, PI 346307 and PI 235346 had resistant reactions in the non-inoculation trial in 2009, but susceptible reaction in both non-inoculated and inoculated tests in 2012. PI 80479 was the most susceptible entry in the tests in 2012, but only mildly susceptible in 2009 [12]. The exact cause of the different reaction to PSD in those soybean entries is unknown, but one of the possible explanations of these inconsistent field results could be due to the difference of the pathogen population in 2009 and 2012. Reaction of certain soybean genotype to different isolates of the pathogen could be different. Therefore, further experiments are needed to test soybean genotypes with multiple isolates.

In addition, results from this study indicated that there was no significant (*P* = 0.4039) difference of stem lesion length caused by *P. longicolla* at 24 °C and 30 °C. Although these two temperatures have been found to be good for the pathogen growth and plant infection, further studies will need to be conducted to test the effect of temperatures under 24 °C and above 30 °C on the stem infection by *P. longicolla*.

In the past decades, many greenhouse and laboratory inoculation methods have been developed to evaluate soybean for resistance to various diseases, such as Phytophthora root rot [48], Sclerotina stem rot [49], and sudden death syndrome (SDS) [50, 51]. In some instances differential reactions of a susceptible (‘Amsoy 71’) and a resistant (‘PI 80837’) soybean line to pod inoculations in greenhouse trials have been reported [52]. This approach might also be useful for screening for resistance to seed infection by *P. longicolla*. Alternatively, Zhang and Xue [53] reported the use of excided leaves to evaluate soybean cultivars for resistance to *P. longicolla* and *S. sclerotiorum* but the use of this method required more tests as there were no resistant and susceptible controls for comparison. Some other previous studies have shown inconsistent results for resistance to *S. sclerotiorum* using the excised leaves method [54, 55]. Unlike these other approaches to assess disease, the cut-seedling inoculation technique developed in this study is, to our knowledge, the first use of a seedling assay to evaluate seed disease resistance in soybean. Furthermore, it is also likely that this method could be applied to other seed disease evaluations, not only in soybean but in other crops as well.

The early soybean production system (ESPS) has been developed with the goal to increase yields and reduce irrigation costs [56]. ESPS is commonly used in the mid-southern U.S. However, in this system, soybeans are planted in March or April, and some cultivars mature in July and August when high temperatures and high humidity were conducive to PSD development, which can lead to low seed germination and high levels of PSD incidence [57]. This makes PSD the most economically important seed disease in the southern soybean production area of the U.S. The identification and utilization of sources of resistance to PSD for breeding programs is important, since planting resistant cultivars is the most economical and environmentally friendly strategy of protecting soybean crops from PSD, especially when using the ESPS in southern states.

The sources of PSD-resistance identified in our previous field test and confirmed with the cut-seedling inoculation assays in this study can be used in developing soybean breeding lines or cultivars with resistance to PSD, and for genetic mapping of PSD resistance gene(s). Experiments are underway to phenotype populations to determine the genetics of resistance to PSD, and to develop high yielding soybean with PSD resistance. Recently, the availability of the whole genome sequences of *P. longicolla* [33, 34] has led to identification of pathogenic protein networks in *P*. *longicolla* underlying seed decay of soybean [58]. The cut-seedling inoculation technique may therefore be a useful tool to fast-phenotyping host reactions to pathogen and mutants in studies investigating pathogenicity-related genes and their proteins.

## Conclusion

Development of a fast inoculation and seedling assay will facilitate identification of genotypes with resistance to PSD, one of the most economically important soybean seed diseases. In this study, a rapid cut-seedling inoculation method was developed and utilized to evaluate soybean genotypes for resistance to PSD under controlled conditions. Although PSD is a seed disease of soybean, this study provided rapid evaluation of soybean for reaction to *P. longicolla* at the seedling stage and identification of PSD-resistant genotypes with comparable results from field tests and was achieved without having to wait until the end of the growing season. Furthermore, results of the assay were not influenced by environmental conditions encountered in the field, or by the consequences of uneven inoculums. Finally, it is suggested that the cut-seedling inoculation technique will be useful for the rapid evaluation of PSD-resistance populations for the discovery of genes underlying this resistance as well as high throughput phenotyping of seed diseases at seedling stage for genetics and genomic studies.

## Abbreviations

ANOVA: analysis of variance
APDA: acidified potato dextrose agar
AUGPC: area under growth progress curve
AUDPC: area under disease progress curve
LSD: least significant difference
MG: maturity group
PI: plant introduction
PSD: Phomopsis seed decay
